# Heterogeneity manifestation and formation mechanism of school youth sports activity quality: a multi-case comparative study based on the configurational perspective

**DOI:** 10.3389/fpsyg.2026.1846719

**Published:** 2026-08-28

**Authors:** Yang Liu, Jiesen Yin

**Affiliations:** 1 Department of Physical Education, Changzhou Vocational Institute of Textile and Garment, Changzhou, Jiangsu, China; 2 Department of Physical Education, Wuxi University of Technology, Wuxi, Jiangsu, China

**Keywords:** configuration analysis, exact match, formation mechanism, quality heterogeneity, youth sports

## Abstract

**Introduction:**

Improving school-based youth sports activities is an important objective of health and education policy, yet schools continue to differ markedly in implementation quality.

**Methods:**

This study combined multi-case comparison with fuzzy-set qualitative comparative analysis (fsQCA) to examine how policy, resources, organization, curriculum, teachers, facilities, evaluation, and student experience jointly shape activity quality. Five schools representing resource-driven, organizationally innovative, and resource-constrained contexts were analyzed.

**Results:**

Organizational leadership displayed quasi-necessary characteristics but was not sufficient alone. Eight high-quality and eight low-quality configurations were identified. High-quality outcomes reflected either the effective transformation of resource advantages or organizational compensation under moderate resources, whereas low-quality outcomes arose from weak foundational support or failures in organizational transformation. Student experience played a process-related role in several configurations. Sensitivity tests showed that the main explanatory structure remained broadly stable across the tested settings.

**Discussion:**

School youth sports activity quality is shaped by combinations of conditions rather than by a single factor. Given the five-school case base, the identified pathways should be interpreted as exploratory mechanisms within the examined schools rather than as estimates of their prevalence in a wider population.

## 1 Introduction

The integration of national health and education policies has made the quality of youth sports activities a central concern in school physical education (Tang and Tang, 2025). Recent policies specify requirements for physical fitness, daily activity time, curriculum delivery, teacher development, and school facilities. They also set targets for adolescent fitness compliance and require schools to safeguard regular participation in physical activity (Yinnan et al., 2025; Simwanza, 2026). These measures have shifted school physical education from a routine curricular matter to a broader issue of student development and educational governance. Implementation, however, remains uneven. Schools operating under similar policy mandates differ substantially in resource investment, organizational capacity, curriculum delivery, and outcomes. Such variation suggests that quality is shaped by interacting conditions rather than by a single input. Conventional linear explanations therefore provide only a partial account of why comparable schools produce different results (Wang et al., 2026).

Existing studies follow three main paths. Single-factor research examines teachers, facilities, curriculum provision, policy implementation, or student participation, but it cannot readily explain different outcomes among schools with similar resources (Chen et al., 2025). Multidimensional evaluation integrates inputs, processes, participation, and health outcomes, yet it mainly describes quality levels rather than their formation. Configurational studies address causal complexity, but most focus on individual learning, teacher education, or general organizational performance. School-level interactions among policy, resources, organizational implementation, and student experience remain underexamined.

This study therefore asks how external policy and resources are transformed through school organization into curriculum delivery, student participation, and quality outcomes (Pereira and Lang, 2025). Policy support and resource allocation are treated as external conditions. Organizational leadership, teacher competence, curriculum implementation, facilities, and evaluation constitute the transformation process, while student experience indicates whether these arrangements reach students' development. Multi-case comparison explains how this process operates in different school contexts, and fsQCA identifies condition combinations within the five schools. The contribution lies in specifying cross-level transformation mechanisms and comparing asymmetric high- and low-quality pathways, not in the use of fsQCA alone (Wang, 2025).

**Figure 1:**
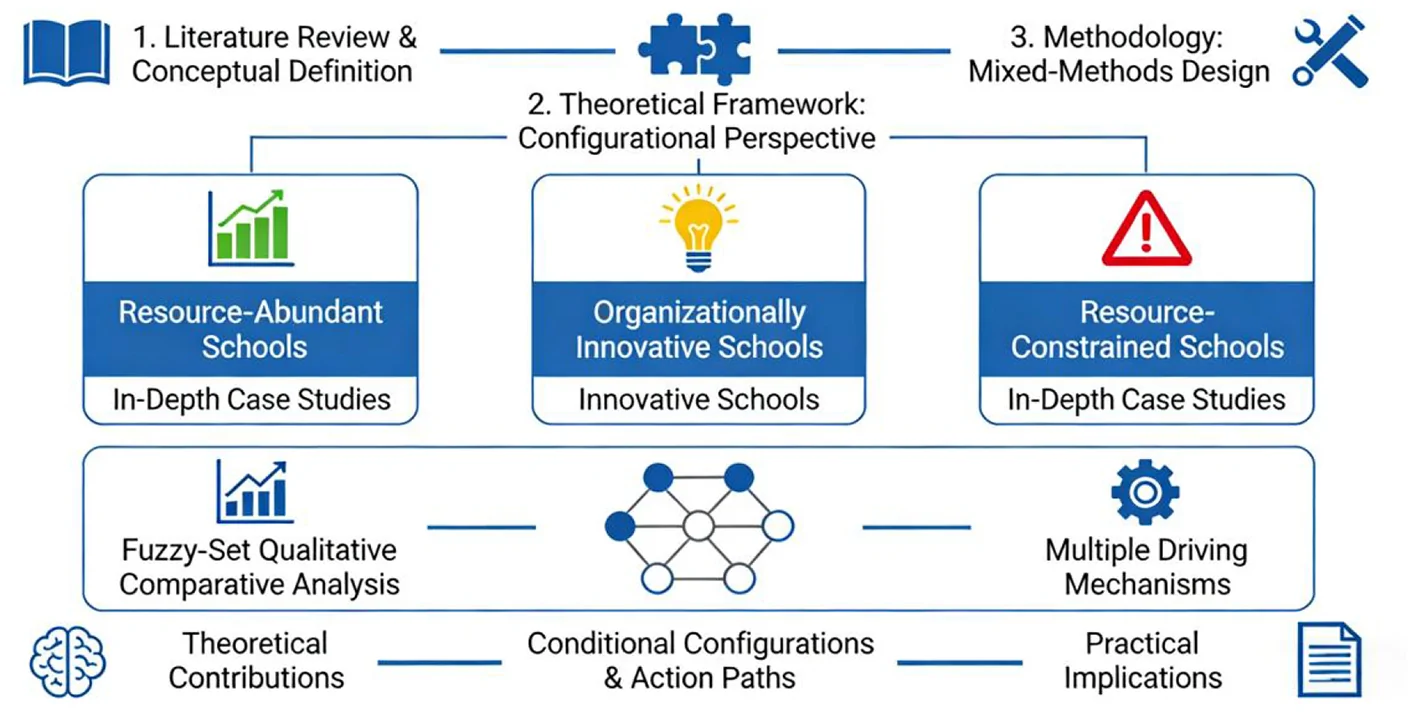
Article structure diagram.

Four questions guide the study. First, how does sports activity quality vary across resource and organizational contexts? Second, which condition combinations support high-quality outcomes? Third, do low-quality outcomes arise through mechanisms distinct from the absence of high-quality conditions? Fourth, how does student experience interact with organization, resources, and curriculum implementation? The study first defines the core concepts and theoretical framework, then combines case comparison with configurational analysis, and finally interprets the results and their practical implications (Venkatraman et al., 2022; Zou et al., 2025; Teare and Taks, 2025; O'Neil and Smith, 2026). Figure 1 summarizes this research sequence.

## 2 Literature review and research framework

### 2.1 Operational definition of core concepts

This study examines two core concepts: youth sports activity quality and quality heterogeneity (Hosokawa et al., 2026). Activity quality is operationalized through the CIPP dimensions of context, input, process, and outcome. These dimensions capture policy and strategic support, teachers and facilities, curriculum organization and student experience, and gains in health, skills, and interest (Ashraf et al., 2025; Ma, 2025).

Heterogeneity refers to systematic differences in how schools achieve quality under different internal and external conditions (He et al., 2025). The analysis distinguishes resource-driven schools, organizationally innovative schools that improve resource use through management, and resource-constrained schools facing persistent foundational limitations (Zhu et al., 2025).

### 2.2 Review of relevant research at home and abroad

Early research on school physical education concentrated on resource inputs and instructional conditions, including teacher staffing, facilities, curriculum time, activity provision, and policy implementation. These studies identify important constraints but generally estimate the average effect of individual factors (Won et al., 2025). They therefore offer limited explanation for schools that achieve different outcomes with similar resources or maintain effective curriculum delivery despite resource constraints.

A second research stream evaluates school physical education through multidimensional indicators covering context, input, process, and outcome (Zou and Liu, 2025). This approach improves the description of school quality but focuses mainly on indicator selection, weighting, and performance differences. Composite scores show whether quality is high or low, yet they do not reveal how resources, organizational capacity, and student experience combine or compensate for one another.

Recent international education research has used fsQCA to examine classroom participation, organizational operation, and learning outcomes. These studies show that similar outcomes may arise from different condition combinations and that a condition's relevance depends on its configuration. However, most work concerns individual learning, teacher education, or general performance, with limited integration of school-level resource transformation and student experience. Sport psychology research has separately emphasized motivation, interest, competence, satisfaction, and continued participation. Student experience is often modeled as a predictor, mediator, or outcome rather than as evidence that policy, resources, curriculum, and teaching have been translated into meaningful participation (Espedalen and Seippel, 2025). This leaves a gap between student-level experience and school-level institutional processes.

**Figure 2:**
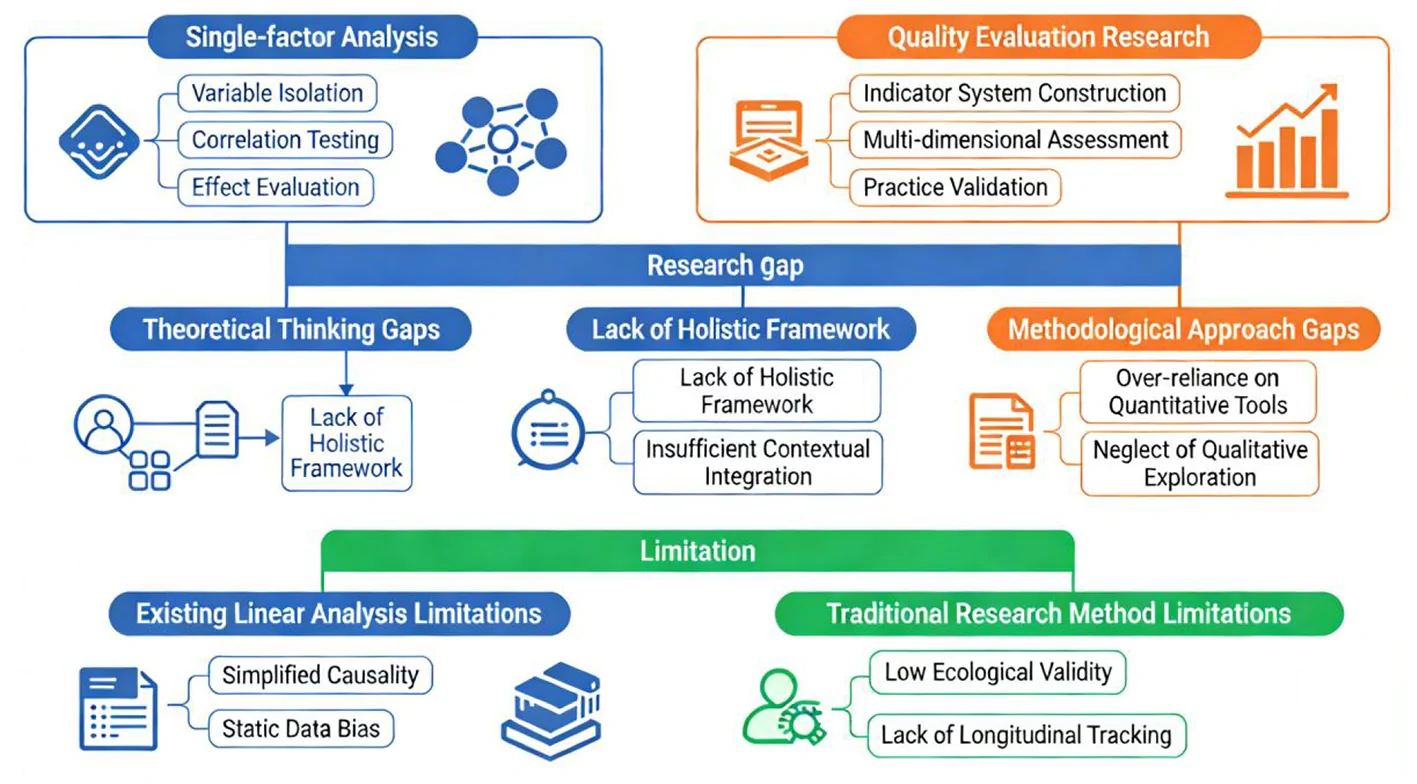
Domestic and foreign research model diagram.

The present study addresses this gap by examining three issues: whether policy and resources require organizational and curricular transformation to generate quality, whether organization and positive student experience can compensate for moderate resources, and whether low quality reflects distinct failure mechanisms. Social-ecological systems theory, the CIPP framework, case evidence, and fsQCA are integrated into an exploratory cross-level model shown in Figure 2.

### 2.3 Theoretical basis and construction of analytical framework

Social-ecological systems theory explains development as an outcome of interactions across environmental levels. In school physical education, policy and resources create opportunities but do not directly produce student outcomes. They must be translated through organizational leadership, teacher implementation, curriculum delivery, and student participation (Svacina et al., 2025). At the macro level, policy provides institutional direction and resources. At the school level, leadership coordinates departments, allocates resources, supports teachers, and monitors implementation. Teacher competence, curriculum quality, facilities, and evaluation then determine whether external support becomes routine practice (Guernon et al., 2025). Organizational leadership therefore functions both as a condition and as a link between policy and implementation.

At the student level, subjective experience captures interest, satisfaction, perceived gain, and willingness to continue participating. Positive experience depends on accessible content, stable delivery, and effective organization rather than on resources alone (Shi, 2025). It may complement organizational innovation in resource-limited settings, while poor experience may reduce the value of otherwise strong resources.

Accordingly, school sports activity quality is treated as a cross-level transformation outcome. Policy and resources define what a school can access; organization, teachers, curriculum, facilities, and evaluation determine how those assets are used; student experience indicates whether they produce participation and development (Meiner et al., 2025). CIPP defines the four quality dimensions, while the configurational perspective identifies alternative combinations across schools (Skolmowska et al., 2024).

The framework differs from additive models by treating external support, organizational transformation, and student feedback as an interdependent system. High quality may result from converting strong resources into effective practice or from organizational innovation that compensates for moderate resources. Low quality may reflect either weak foundational support or failed transformation (Mello et al., 2025). Recent experimental evidence indicates that improvised physical activity can enhance divergent thinking in adolescents (Jin et al., 2026).

### 2.4 Theory-driven configurational propositions

**Figure 3:**
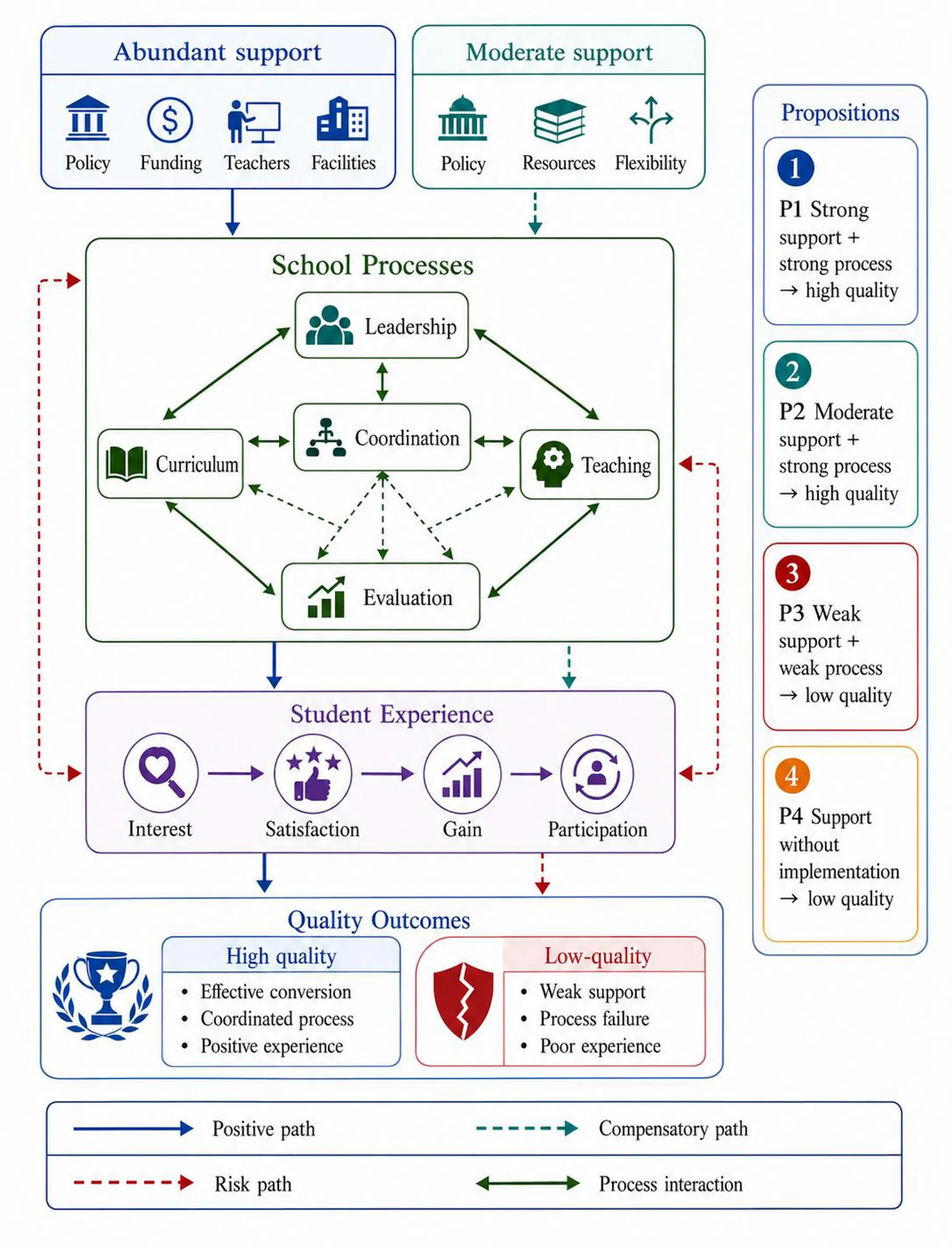
Theory-driven configurational propositions for school sports activity quality.

Social-ecological systems theory and CIPP imply that resources affect quality through organizational and instructional processes. In resource-abundant schools, policy support, funding, teachers, and facilities provide development capacity, but high quality also requires coordination, stable curriculum implementation, and professional teaching (Cheng et al., 2024). Resource advantages therefore create opportunity, while school processes determine whether that opportunity becomes educational value (Figure 3).

Schools with moderate resources may rely more on internal coordination, curriculum adaptation, teacher collaboration, facility scheduling, and evaluation feedback. Positive student experience indicates whether these arrangements generate interest, satisfaction, perceived gain, and continued participation. Low quality may arise either from simultaneous weakness in policy, resources, staffing, and facilities or from a failure to translate available support through organization, curriculum, evaluation, and participation (Miquel-Romero et al., 2024).

The following propositions guide condition selection and interpretation. They are exploratory expectations for the five cases rather than statistical claims about the wider school population.

P1: Resource-abundant schools are more likely to achieve high quality when policy support and resource allocation are combined with strong organizational leadership, stable curriculum implementation, and teacher competence.

P2: Under moderate policy or resource conditions, organizational leadership, curriculum quality, teacher competence, and positive student experience may form a compensatory high-quality configuration.

P3: Low quality is more likely when weak policy, leadership, and resources coincide with deficiencies in curriculum implementation, teacher competence, or facilities.

P4: Even where some external support exists, low quality may persist when organization, curriculum implementation, evaluation feedback, or student experience fails to convert that support into practice.

## 3 Research design and methodology

### 3.1 Research paradigm and mixed methods design

This study employs a mixed-methods research design that combines multi-case comparison with fsQCA (fuzzy-set Qualitative Comparative Analysis) as a structured comparative tool. Rather than analyzing two independent sets of samples separately, both methods are applied to the same group of five schools. Multi-case comparison is primarily used to identify contextual differences regarding school resources, organizational operations, curriculum implementation, and student engagement experiences, while fsQCA is used to compare the configurational relationships formed by these conditions at the school level. The study aims not to estimate the average net effect of a single variable, but to examine how various conditions combine within specific school contexts to lead to higher or lower quality in physical activity.

The research evidence is derived from student questionnaires, questionnaires and interviews with school personnel, on-site observations, school archives, and existing evaluation records. Different data sources serve distinct functions in the analysis. School archives and on-site observations are primarily used to assess relatively objective conditions such as funding, teachers, venues, facilities, and curriculum implementation. Questionnaires are mainly used to measure conditions requiring participant evaluation, such as organizational leadership and students' subjective experiences. Interview data are used to explain institutional arrangements, inter-departmental coordination, resource utilization, and the curriculum execution process. Expert reviews are employed to verify whether the indicator selection, content coverage, and weighting align with the theoretical conceptualization of physical activity quality in schools (Mills and Fort, 2023).

Qualitative and quantitative evidence are integrated across three stages. The first stage is variable formation; both condition variables and outcome variables are determined through a combination of the theoretical framework and multi-source data, rather than being generated directly from a single questionnaire. The second stage involves assessment at the school level; questionnaire results, archival indicators, observation records, and interview data are cross-referenced to verify the actual status of each school regarding specific conditions, thereby avoiding reliance on a single information source to determine calibration parameters. The third stage is the interpretation of results; fsQCA is used to identify potential condition configurations within the sample, while multi-case data illustrate how these configurations emerge in different school contexts and why they result in varying levels of quality.

This study treats the five schools as a theory-driven, small-scale comparative sample. Case selection emphasizes typological diversity, data completeness, and contextual comparability rather than statistical representativeness. Consequently, the fsQCA results serve primarily to explore configurational relationships within the sample; they are not used to infer the prevalence of specific pathways across the broader population of schools, nor are the identified pathways interpreted as universally applicable causal laws. The limited number of cases may result in insufficient configurational diversity and path overlap; consequently, the subsequent analysis reports consistency, raw coverage, unique coverage, and sensitivity tests, while the conclusion limits the scope of extrapolation (Xu et al., 2023).

In the data processing stage, the study employs the fuzzy set qualitative comparative analysis method to calibrate and transform variables (Wang et al., 2022). The fuzzy set calibration function converts the original continuous variables into membership scores ranging from zero to one, achieving qualitative processing of quantitative data. This function ensures comparability of data across different cases by setting three calibration anchor points: complete membership, intersection point, and complete non-membership. Equation 1 shows its mathematical expression.

$$\begin{array}{l} \mu \left(x\right)=\frac{1}{1+e^{-k\left(x-c\right)}} \end{array}$$ (1)

μ(*x*) It represents the membership degree, *x* is the original variable value, *c* is the calibration anchor point, *k* and is the slope parameter. This function converts continuous variables into fuzzy set scores within the [0, 1] interval, enabling qualitative processing of quantitative data.

Once the three anchor points were established, a direct calibration method was employed to calculate the set membership scores for the cases. Cases situated at the crossover point exhibit the highest level of uncertainty regarding set membership; consequently, they cannot be simply classified as “average-level” schools. To avoid the potential impact of scores falling exactly at the 0.50 mark on truth table analysis, scores near the crossover point were handled according to a standardized rule; the raw scores, standardized scores, anchor points, and final membership scores for the five schools are reported in the supplementary materials.

The core of configuration analysis lies in examining the set relationship between condition combinations and outcomes. The consistency coefficient is used to measure the explanatory power of a certain condition combination on the outcome. The higher the coefficient, the stronger the reliability of the combination as a sufficient condition. This coefficient is obtained by calculating the ratio of the sum of the minimum membership degrees of cases in the condition combination and outcome to the total membership degree of the condition combination. Equation 2 shows its calculation method.

$$\begin{array}{l} Consisteny=\frac{\sum \min\left(X_{i},Y_{i}\right)}{\sum x_{i}} \end{array}$$ (2)

*X*_*i*_ represents the membership degree of condition combinations, *Y*_*i*_ and represents the membership degree of outcomes. This coefficient measures the explanatory power of condition combinations on outcomes, with a value range of [0, 1]. A higher value indicates stronger sufficiency.

**Figure 4:**
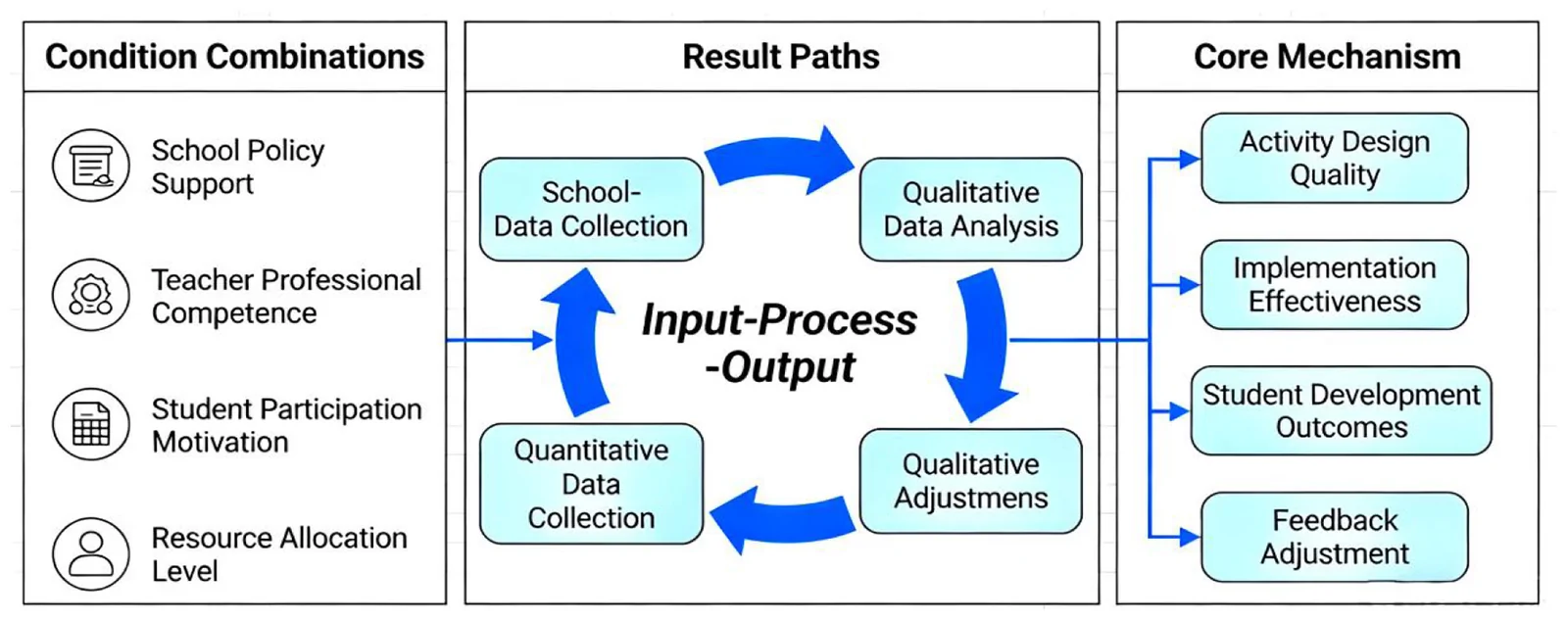
Mixed methods design model diagram.

Through the aforementioned hybrid method design, the study has achieved a leap from deep description of multiple cases to configurational pattern recognition, laying a methodological foundation for subsequently revealing the heterogeneous formation paths of sports activity quality (Figure 4).

### 3.2 Case selection and sample description

The case selection of this study follows the principle of combining purposive sampling with maximum difference sampling. In order to avoid confusing the types of schools with the number of cases, this paper clearly defines the analysis unit as schools, and finally includes five sample schools, including two resource driven schools, two organizational innovation schools and one resource constrained school. The case a, case B and case C used in Section 4 do not represent three independent sample schools, but a typological summary of the formation mode of the quality of sports activities in the three types of schools. Among them, case a corresponds to a resource driven school, which is mainly used to present the typical characteristics of improving the quality of sports activities under the condition of sufficient resources; Case B corresponds to an organization innovative school, which is mainly used to present the process of improving quality through management innovation under the condition of medium resources; Case C corresponds to a resource constrained school, which is mainly used to present the realistic situation of limited quality and partial improvement of sports activities when the basic conditions are insufficient.

The selection of sample schools is mainly based on four criteria. First, the school has clear type characteristics in resource allocation level, organization and management mode and sports activity quality results, which can represent three development scenarios: resource driven, organizational innovation and resource constraint. Second, there are obvious differences in the regional location, school size, sports funding, teacher allocation and site conditions of schools, which can provide an empirical basis for comparing the quality formation mechanism under different conditions. Third, the school can provide relatively complete questionnaires, interviews, field observations and archival data to ensure that variable calibration and case interpretation have traceable data sources. Fourth, the samples are not only different, but also basically comparable in the implementation framework of school sports activities, which can support the subsequent configuration comparative analysis.

The unit of analysis in this study is the school; all five sample schools were included in both the multi-case comparison and the fsQCA. The multi-case comparison serves to illustrate contextual differences among the schools regarding resource bases, organizational approaches, and quality performance, while the fsQCA is used to compare set-theoretic relationships across the same group of schools based on eight conditions. Cases A, B, and C, as presented in Chapter 4, are not additional, separate cases; rather, they are typological narratives constructed from the shared characteristics of the five schools. Case A encapsulates the primary mechanisms of resource-driven schools; Case B, those of organizationally innovative schools; and Case C, those of resource-constrained schools.

The selection of the five schools was guided by theoretical contrast and the principle of maximum variation, aiming to encompass three distinct school contexts: resource-abundant, resource-moderate, and resource-constrained. While this design facilitates an in-depth cross-case comparison of mechanisms, it does not provide statistical representativeness of the broader population. Consequently, the resulting configurations should be interpreted as exploratory patterns specific to these five schools. The case coverage figures in Tables 1, 2 indicate the extent of the sample explained by a specific combination of conditions; as a single school may be covered by multiple pathways, the coverage counts across pathways cannot be summed, nor can they be used to infer the frequency of these pathways among schools in the wider population in Table 3.

**Table 1:** Detailed list of high-quality path configurations.

Condition variable	Path 1	Path 2	Path 3	Path 4	Path 5	Path 6	Path 7	Path 8
Policy support intensity			⊗			⊗		
Organizational leadership		⊗					⊗	
Resource allocation level				⊗				⊗
Students' subjective experience	⊗							
The quality of curriculum implementation					⊗			
Professional level of teachers		⊗						
Level of facility perfection	⊗					⊗		
Scientificity of evaluation mechanism							⊗	
Consistency	0.934	0.926	0.919	0.928	0.922	0.917	0.925	0.92
Original coverage	0.325	0.298	0.286	0.312	0.275	0.264	0.301	0.289
Unique coverage	0.078	0.065	0.052	0.071	0.048	0.041	0.068	0.055
Covered case count	4	3	3	4	3	2	4	3

Symbol explanation: indicates the presence of a condition, while ⊗ indicates the absence of a condition. The core and edge conditions are determined by comparing the reduced solution with the intermediate solution. In order to avoid excessive complexity of symbols, the main presentation conditions in the table are existence and absence. See the text for the explanation of core conditions and edge conditions.

**Table 2:** Detailed list of low-quality path configurations.

Condition variable	Path 1	Path 2	Path 3	Path 4	Path 5	Path 6	Path 7	Path 8
Policy support intensity	⊗	⊗		⊗	⊗		⊗	⊗
Organizational leadership	⊗		⊗	⊗	⊗	⊗		⊗
Resource allocation level	⊗	⊗	⊗		⊗	⊗	⊗	
Students' subjective experience		⊗	⊗	⊗		⊗	⊗	⊗
Course implementation quality	⊗	⊗	⊗	⊗	⊗		⊗	⊗
Professional level of teachers	⊗	⊗		⊗	⊗	⊗	⊗	⊗
Degree of facility perfection		⊗	⊗	⊗	⊗	⊗		⊗
Scientificity of evaluation mechanism	⊗		⊗	⊗		⊗	⊗	
Consistency	0.918	0.925	0.912	0.92	0.916	0.922	0.919	0.914
Original coverage	0.312	0.285	0.278	0.296	0.268	0.302	0.291	0.275
Unique coverage	0.071	0.062	0.055	0.068	0.051	0.073	0.065	0.058
Covered case count	4	3	3	4	3	4	3	3

Symbol explanation: indicates the presence of a condition, while ⊗ indicates the absence of a condition.The core and edge conditions are determined by comparing the reduced solution with the intermediate solution. In order to avoid excessive complexity of symbols, the main presentation conditions in the table are existence and absence. See the text for the explanation of core conditions and edge conditions.

**Table 3:** Basic characteristics and sampling information of the case schools.

School type	Number	Regional	Size	Ratio of physical education teachers	Annual sports expenditure per student	Area	Quality assessment
Resource-driven	SCH-A01	Eastern provincial capital	2,500	0.180555556	1,200	8.5	92.3
Organizational	SCH-B01	Cities in the central region	1,800	0.194444444	850	6.2	88.7
Resource-constrained	SCH-C01	Western county regions	950	0.236111111	480	4.1	76.5
Organizational	SCH-B02	Eastern county	1,200	0.1875	780	5.8	86.9
Resource-driven	SCH-A02	eastern coastal areas	3,000	0.173611111	1,500	9.2	94.1

This systematic presentation of data holds significant importance. By quantitatively demonstrating the disparities among three types of schools in terms of regional distribution, resource allocation, and quality outcomes, it provides an empirical foundation for understanding the heterogeneous performance of school physical activity quality. The data distribution in the table intuitively reflects the potential correlation between resource allocation and quality outcomes, while also suggesting the existence of different paths for quality improvement. For instance, although resource-constrained schools have an overall inadequate investment, they still exhibit a quality performance of 76.5 points, indicating that factors other than resource allocation may have a significant impact on quality improvement. These findings directly echo the exploration of heterogeneous performance and formation mechanisms in the research topic, laying a data foundation for subsequent configurational analysis.

### 3.3 Variable measurement and data sources

The measurement model distinguishes reflective constructs from school-level condition indicators. Organizational leadership and student subjective experience were measured with multi-item scales and assessed for internal consistency, composite reliability, and convergent validity. Policy support, resource allocation, curriculum implementation, teacher competence, facility adequacy, and evaluation quality were formed from records, observations, evaluation materials, questionnaires, and interviews. For these indicators, transparency of definitions, sources, scoring, and cross-school comparability was more appropriate than internal-consistency testing (Table 4).

**Table 4:** Variable definitions and measurement methods.

Variable type	Variable name	Operational definition	Measurement method	Data source	Calibration anchor point
Result	Quality of sports activities	Comprehensive index based on the four dimensions of the CIPP model and expert-reviewed weighting	Weighted comprehensive evaluation	Field observation and document analysis	0.95/0.5/0.05
Condition	Policy support intensity	Special funds and the number of policy documents	quantitative statistics	Archives of the Education Department	0.8/0.5/0.2
Condition	organizational leadership	Principal leadership and management system perfection	Likert 7-point scale	In-depth interviews and questionnaires	0.8/0.5/0.2
Condition	Resource allocation level	Comprehensive evaluation of teachers, venues, and equipment	Objective indicator measurement	field research	0.8/0.5/0.2
Condition	Students' subjective experience	Participation interest and satisfaction evaluation	Likert 7-point scale	Student questionnaire survey	0.8/0.5/0.2

The outcome was a CIPP-based index of context, input, process, and outcome quality. These dimensions captured policy and institutional support, funding and facilities, curriculum and participation, and student fitness, skills, interest, and sustained participation. Expert-reviewed indicators were combined with observations, archives, evaluation records, and questionnaires. The measurement structure mirrors the theoretical model: external support defines available capacity, school-level conditions represent transformation, and student experience reflects whether that capacity reaches participation and development.

Curriculum implementation and student experience were related but distinct. The former measured curriculum coverage, activity organization, teaching standards, and implementation stability. The latter measured interest, satisfaction, perceived gain, and willingness to continue. Item review, expert assessment, and cross-source comparison reduced overlap.

Organizational leadership captured the place of physical education in school planning, management-system stability, departmental coordination, and support for teachers. Student experience captured interest, satisfaction, perceived gain, and continued participation. Each construct used five items rated on a seven-point scale.

Five experts reviewed wording, theoretical relevance, content coverage, and contextual applicability. Organizational leadership showed Cronbach's alpha of 0.82, composite reliability of 0.85, and AVE of 0.58. Student experience showed corresponding values of 0.84, 0.87, and 0.61, supporting acceptable internal consistency and convergent validity.

Interview and observation materials were categorized using the eight conditions and four quality dimensions. Their role was to cross-check school-level judgments and explain differences, not to convert theme frequency directly into fuzzy scores. Records and observations verified objective conditions, questionnaires provided structured assessments, and interviews clarified organizational and curricular mechanisms. Conflicting evidence was rechecked against the original sources.

Curriculum implementation described the school-side process, whereas student experience described students' response to that process. Maintaining this distinction was essential for interpreting organizational compensation and transformation failure.

The CIPP outcome combined expert-reviewed indicators and weights with observations, records, evaluation logs, and questionnaire data, providing a traceable basis for calibration.

### 3.4 Data processing and fsQCA analysis process

Before calibration, raw school-level scores were calculated under the specified scoring rules and standardized to a zero-to-one scale. Standardization addressed different measurement units but did not determine set membership. Anchors were then selected from theoretical meaning, scale semantics, external standards, and expert-validated quality levels.

Anchors were not based solely on the five-school sample's maxima, minima, or quantiles, which would be unstable in a small sample. External school-sport standards guided indicators with explicit benchmarks. Seven-point constructs used thresholds representing clearly high, ambiguous, and clearly low states. Operational definitions and expert-validated scoring intervals guided indicators without uniform external standards.

The outcome used standardized anchors of 0.95, 0.50, and 0.05 to distinguish near-complete CIPP attainment, maximum ambiguity, and clear non-attainment. Condition variables used 0.80, 0.50, and 0.20 to represent clear presence, ambiguity, and clear insufficiency. These were pre-calibration thresholds rather than assigned membership scores.

Subsequently, the study employed the Boolean minimization algorithm to simplify the combination of conditions and identify the core condition combinations that could explain the results. This algorithm eliminates redundant conditions through logical operations, retains the necessary factors that constitute a sufficient relationship, and thus obtains the simplest solution. Equation 3 presents the mathematical expression of this process.

$$\begin{array}{l} Outcome=\vee_{p=1}^{p}\left(\wedge_{q-1}^{Q_{P}}Condition_{pq}\right) \end{array}$$ (3)

*Outcome* Represents the outcome variable, *Condition*_*pq*_ and denotes the th condition in the th combination.

Finally, the interpretation scope of each condition combination on the results is evaluated through coverage measurement, which measures the importance level of different paths. The calculation of coverage is based on the membership relationship between condition combinations and result sets. The higher the value, the greater the proportion of cases explained by that path. Equation 4 specifically illustrates its calculation method.

$$\begin{array}{l} Coverage=\frac{\sum \min\left(X_{i},Y_{i}\right)}{\sum Y_{i}} \end{array}$$ (4)

*X*_*i*_ Represents the membership degree of the condition combination, *Y*_*i*_ and represents the membership degree of the result.

The calibration strategy combined theory, external standards, expert review, and case evidence. It avoided mechanical reliance on sample extremes and treated the crossover as maximum ambiguity rather than an average state.

**Figure 5:**
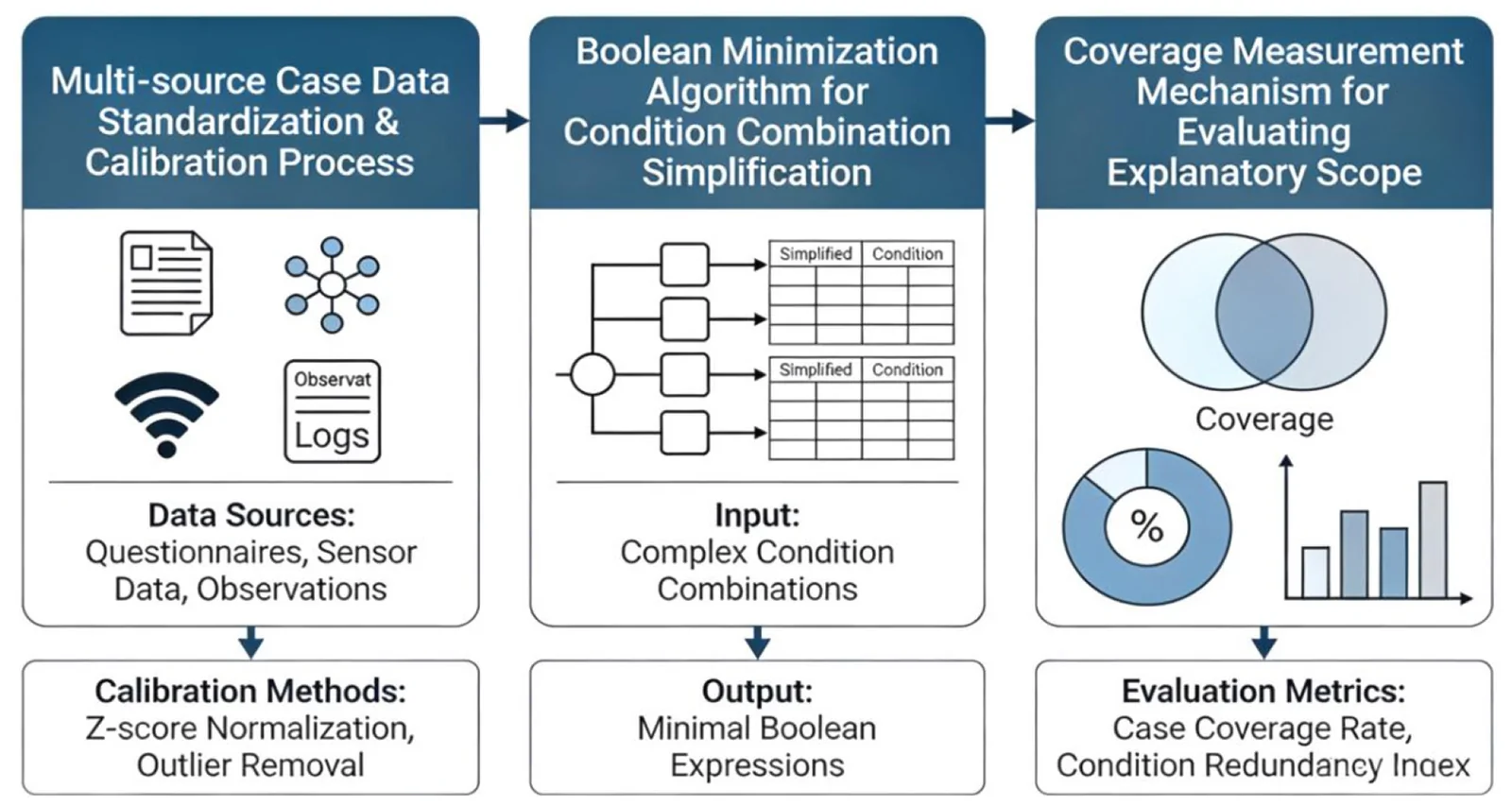
Data processing and analysis model diagram.

After calibration, a truth table was constructed with a frequency threshold of 1, consistency threshold of 0.85, and PRI threshold of 0.75. Unsupported logical combinations were used in the intermediate solution only when consistent with theoretical expectations. Interpretation emphasized the intermediate solution and reported consistency, raw coverage, and unique coverage. The analysis identifies within-sample configurations rather than their population prevalence (Figure 5).

## 4 Case presentation: heterogeneity of sports activity quality

### 4.1 Case A: characterization of resource-driven high-quality model

Case A represents the resource-driven model. Located in an economically developed eastern coastal area, it had strong policy support, high per-student expenditure, well-equipped facilities, and professionally trained teachers. Its teacher-student ratio exceeded the national benchmark, and the school implemented national curriculum requirements alongside distinctive activities such as sailing and golf. These conditions supported strong teaching, activity organization, and competition performance.

However, upon closer observation, it is found that the success of the resource-driven model does not solely rely on the superiority of material conditions. The school excels in resource utilization efficiency, achieving maximum resource benefits through scientific management. The leadership attaches great importance to sports work, establishing a comprehensive organizational management system to ensure that various resources are precisely invested in key areas. The teaching team not only possesses professional expertise but also demonstrates a high degree of teaching enthusiasm and innovative consciousness, capable of fully utilizing existing resources to develop distinctive courses and activity plans. Furthermore, the school focuses on creating a positive sports culture atmosphere, stimulating students' participation enthusiasm and interest in sports through the organization of various sports competitions and activities. This ability to transform resource advantages into educational benefits has enabled School A to maintain a leading position in terms of high-quality output, providing an important reference for understanding the relationship between resource conditions and quality output.

### 4.2 Case B: quality improvement model driven by organizational innovation

Case B achieved high-quality outcomes with moderate funding and space through organizational innovation. A flat management structure expanded teacher autonomy, while modular and project-based courses, flexible scheduling, and cross-disciplinary links with health and arts education increased responsiveness to student needs. The case demonstrates how management can improve the use of ordinary resources.

The school excels in resource utilization efficiency, maximizing the use of limited resources through meticulous management. The physical education teacher team actively conducts teaching research and innovatively develops multiple low-cost and high-benefit sports activities, such as the transformation of traditional games and the development of simple equipment. The school has also established a home-school-community collaboration mechanism, integrating community sports resources and parent volunteer efforts, expanding the space for sports activities and sources of teachers. In terms of evaluation mechanisms, the school has constructed a diversified sports evaluation system, focusing not only on sports skills and physical fitness levels but also on students' participation experience and the cultivation of healthy behaviors. This innovation-oriented development model provides a practical example for schools in medium-resource areas to improve sports quality, highlighting the core value of organizational innovation in the process of quality improvement.

### 4.3 Case C: quality dilemma model under resource constraints

Case C illustrates the constraints created by weak foundational conditions. Located in a western county, it faced low expenditure, limited activity space, inadequate equipment, and a shortage of specialist physical education teachers. Some classes were taught by teachers from other subjects. These limitations reduced curriculum stability and restricted distinctive or extracurricular activities.

Despite facing the challenge of resource constraints, School C in the case study has attempted to alleviate the quality dilemma through limited innovation. The school has implemented measures such as staggered use of venues to improve the utilization efficiency of existing facilities. In terms of teaching staff, it has enhanced the professional abilities of part-time teachers through school-based training and regional teaching and research activities, while also tapping into local traditional sports resources and developing sports activities with regional characteristics. Furthermore, the school emphasizes the improvement of basic teaching quality, ensures the implementation of national curriculum standards, and guarantees students' basic sports activity needs under limited conditions. Although these efforts cannot fundamentally change the current situation of resource constraints, they have alleviated the trend of quality decline to some extent. They provide an important reference for understanding the development path of school sports in a resource-constrained environment and reflect the practical wisdom of grassroots schools in seeking breakthroughs in difficult situations.

### 4.4 Cross-case comparison and preliminary exploration of heterogeneity

**Figure 6:**
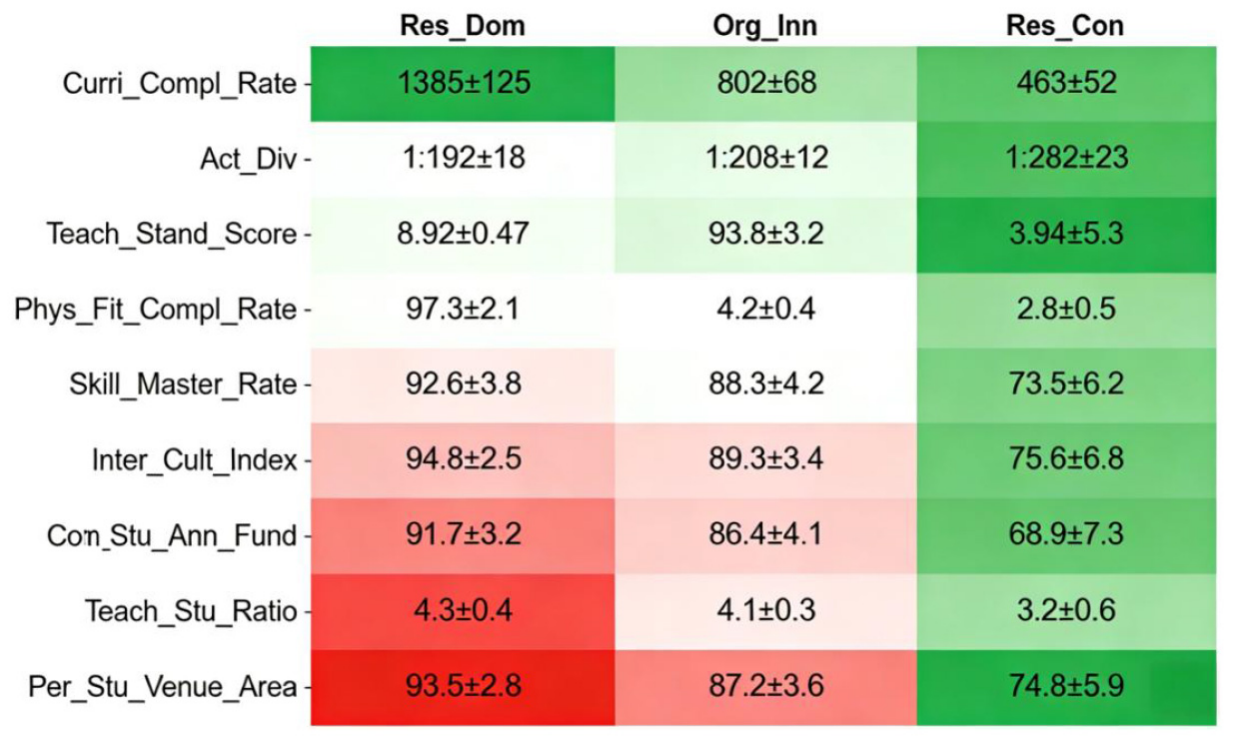
Multi-dimensional comparative analysis of activity quality.

Table 5 shows a clear gradient across the three school types. Resource-driven schools exceeded national standards and achieved a comprehensive score of 93.5. Organizationally innovative schools approached or met most standards despite moderate inputs and scored 87.2. Resource-constrained schools lagged particularly in implementation and outcomes, with a score of 74.8. Differences within each type also indicate that resources alone do not determine quality (Figure 6).

**Table 5:** Multi-dimensional comparative analysis of the quality of school sports activities.

Evaluation dimension	Measurement metrics	Resource-driven	Organizational innovation	Resource-constrained	National standard
Resource input	Average expenditure per student	1385 ± 125	802 ± 68	463 ± 52	≥800
	Teacher ratio	1:192 ± 18	1:208 ± 12	1:282 ± 23	≤ 1:220
	Floor area	8.92 ± 0.47	5.87 ± 0.32	3.95 ± 0.28	≥6.0
>Process implementation	Course enrollment rate	97.3 ± 2.1	93.8 ± 3.2	76.4 ± 5.3	≥90
	Activity diversity	4.5 ± 0.3	4.2 ± 0.4	2.8 ± 0.5	≥3.5
	Teaching normativity	92.6 ± 3.8	88.3 ± 4.2	73.5 ± 6.2	≥80
Output effect	Physical fitness compliance rate	94.8 ±2.5	89.3 ± 3.4	75.6 ± 6.8	≥85
	Skill mastery rate	91.7 ± 3.2	86.4 ± 4.1	68.9 ± 7.3	≥80
	Interest cultivation index	4.3 ± 0.4	4.1 ± 0.3	3.2 ± 0.6	≥3.5
Comprehensive quality evaluation	Comprehensive quality score	93.5 ± 2.8	87.2 ± 3.6	74.8 ± 5.9	≥85

The comparison establishes the empirical basis for the configurational analysis. It shows both the importance of resources and the role of organizational management in converting similar inputs into different outcomes. Case A illustrates resource transformation, Case B organizational compensation, and Case C the limits of adaptation under weak foundational support.

## 5 Configuration analysis: formation mechanism of quality heterogeneity

### 5.1 Univariate necessity test

**Figure 7:**
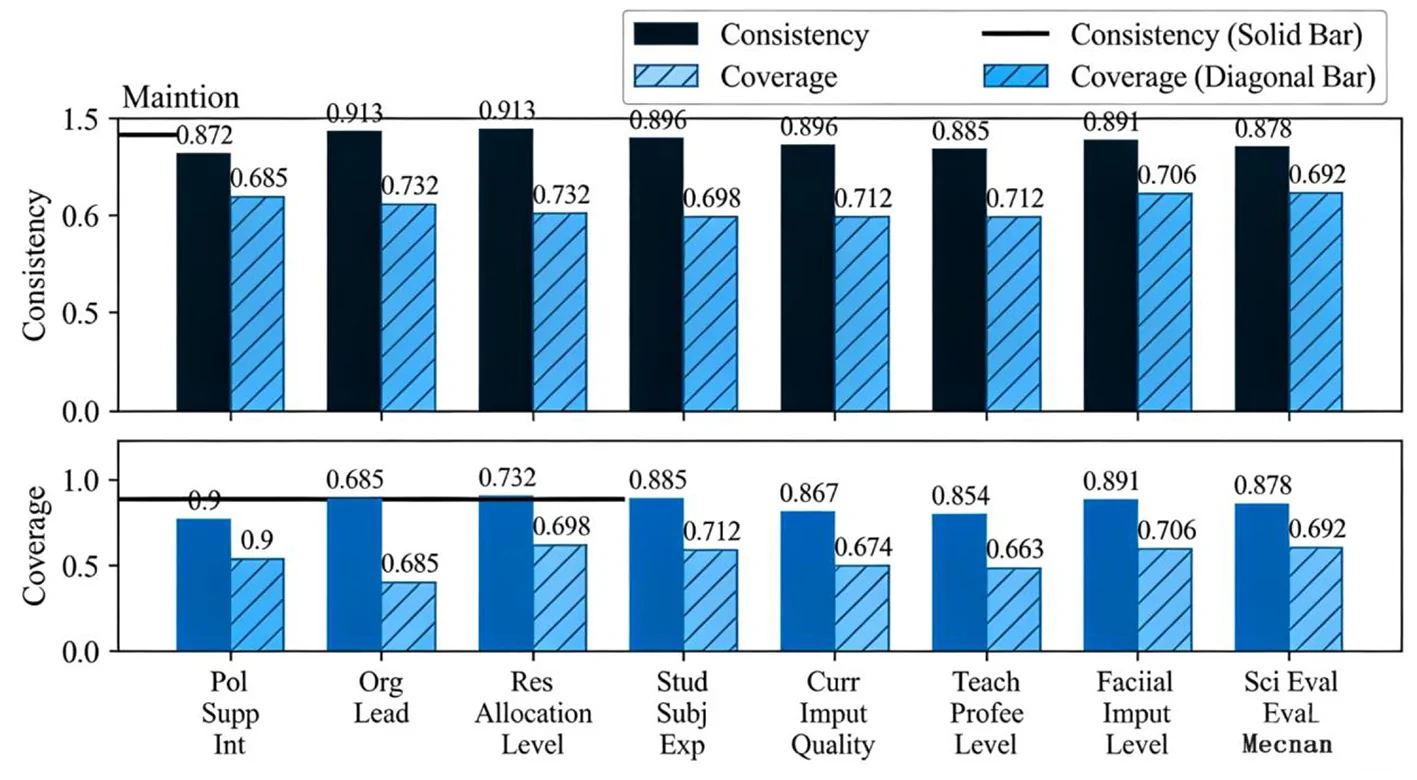
Comparison chart of necessity analysis results.

Necessity analysis examined whether any single condition was required for high quality. Consistency near 0.90 was treated cautiously as evidence of necessary-condition characteristics and interpreted with coverage and theory. Organizational leadership had consistency of 0.913 and coverage of 0.732, indicating quasi-necessary background status. It commonly accompanied high quality but was neither sufficient nor capable of explaining the outcome alone. No other condition reached the 0.90 threshold, as shown in Table 6 and Figure 7.

**Table 6:** Analysis results of the necessity of conditional variables.

Condition variable	Consistency	Coverage
Policy support intensity	0.872	0.685
Organizational leadership	0.913	0.732
Resource allocation level	0.896	0.698
Students' subjective experience	0.885	0.712
The quality of curriculum implementation	0.867	0.674
Professional level of teachers	0.854	0.663
Level of facility perfection	0.891	0.706
Scientificity of evaluation mechanism	0.878	0.692

Necessity analysis is primarily used to determine whether any single condition constrains the occurrence of the outcome, rather than to verify specific research propositions. The consistency score of 0.913 for organizational leadership indicates that high-quality outcomes are typically associated with strong school organizational leadership; thus, it can be cautiously interpreted as a quasi-necessary background condition. However, its coverage is 0.732, and it does not constitute a sufficient explanation on its own. The four configurational propositions still require assessment through a combination of sufficiency analysis and case evidence.

### 5.2 Condition configuration and high-quality path analysis

In the sufficiency analysis stage, this paper constructs a truth table based on the calibrated conditional variables, and uses the truth table as the basis of subsequent Boolean minimization analysis. Considering the sample size and research purpose of this paper, the truth table analysis uses case frequency threshold 1, consistency threshold 0.85 and PRI consistency threshold 0.75 as the main screening criteria. Logical combinations that are not covered by empirical cases are treated as logical residuals and are only used in the analysis of intermediate solutions according to theoretical expectations. In order to enhance the transparency of the report, the complete truth table is listed as supplementary materials. The main body mainly reports the intermediate solution and its corresponding consistency, original coverage and unique coverage.

As shown in Table 7, Overall consistency reflects the extent to which the identified high-quality configurations can be regarded as sufficient conditions for the outcome, while overall coverage indicates the proportion of high-quality cases explained by the solution. The intermediate solution was used as the main basis for interpretation because it balances theoretical plausibility and empirical parsimony.

**Table 7:** Solution parameters of high-quality sports activity configuration.

Solution type	Consistency	Coverage	Unique coverage	Complexity reduction	Simplify the explanation	Theoretical fit	Operability in practice
Overall solution	0.921	0.812	–	0.78	0.82	0.85	0.79
Simple solution	0.935	0.786	0.124	0.85	0.88	0.82	0.83
Intermediate solution	0.928	0.803	0.087	0.82	0.85	0.87	0.81
Complex solution	0.913	0.823	0.056	0.75	0.78	0.89	0.76
Standard solution	0.931	0.795	0.103	0.83	0.86	0.84	0.82

Table 8 distinguishes core from peripheral conditions by comparing parsimonious and intermediate solutions. A large solid symbol denotes core presence, a large crossed symbol core absence, a small solid symbol peripheral presence, a small crossed symbol peripheral absence, and a blank indicates no clear restriction.

**Table 8:** Solution parameters for low-quality sports activity configuration.

Solution type	Consistency	Coverage	Unique coverage	Complexity reduction	Simplify the explanation	Theoretical fit	Operability in practice
Overall solution	0.912	0.783	–	0.76	0.8	0.83	0.77
Simple solution	0.928	0.752	0.118	0.82	0.85	0.8	0.81
Intermediate solution	0.92	0.768	0.092	0.79	0.83	0.84	0.79
Complex solution	0.905	0.791	0.063	0.73	0.76	0.86	0.74
Standard solution	0.924	0.761	0.108	0.81	0.84	0.82	0.8

Table 8 indicates that the five schools did not achieve high-quality outcomes through identical combinations of conditions. Schools with superior resource conditions were characterized primarily by the synergy among policy support, resource allocation, and organizational leadership; this suggests that external resources translate into quality advantages only when mediated by school organization and curriculum implementation. Schools with moderate resource levels relied more heavily on the interplay between organizational leadership, curriculum implementation, and students' subjective experiences, demonstrating how management innovation and process optimization can compensate for a lack of objective resources.

Within the scope of the case studies, these results support the theoretical expectation that quality outcomes can emerge through multiple pathways; however, they do not allow for generalizations regarding the prevalence of these pathways across a broader range of schools. Since a single school may be characterized by multiple pathways simultaneously, the number of pathways listed in the table does not correspond to mutually exclusive school types, nor does it reflect the frequency of these pathways within the general population.

### 5.3 Condition configuration and low-quality path analysis

Table 7 shows that low-quality solutions had slightly lower consistency but comparable coverage to high-quality solutions. Path coverage overlaps across cases and does not represent additional observations. Complexity reduction ranged from 0.73 to 0.82 and explanatory simplicity from 0.76 to 0.85.

Low-quality configurations were derived from the same truth-table and minimization procedures. Table 8 uses the symbol system applied in Table 6 to distinguish core and peripheral conditions. Consistency and coverage indicate acceptable within-sample explanatory relevance, but the configurations remain exploratory because of the limited case base.

The detailed Table 8 of low-quality path configurations presents the specific composition of eight failed paths. Paths 1, 4, and 6 are core paths, each covering 3–4 cases, with consistencies ranging from 0.918 to 0.922. These paths indicate that low-quality results often stem from the simultaneous absence or negative combination of multiple key conditions. For example, Path 1 demonstrates the simultaneous absence of policy support, organizational leadership, and resource allocation, while Path 4 reflects a dual deficiency in resource allocation and student experience. The original coverage ranges from 0.268 to 0.312, and the unique coverage ranges from 0.051 to 0.073, indicating significant differences between the paths.

Low-quality configurations can be categorized into two types of mechanisms: failure of foundational support and failure of organizational transformation. Path 1 is characterized by a simultaneous lack of policy support, organizational leadership, resource allocation, curriculum implementation, teacher professional competence, and evaluation mechanisms; this demonstrates that when multiple fundamental conditions are weak, isolated positive factors are insufficient to sustain high-quality outcomes. This configuration aligns with the constraints regarding funding, teachers, facilities, and curriculum delivery observed in Case C, thereby providing support for Proposition 3 (P3) within the scope of the case schools.

Other paths primarily reflect a failure of organizational transformation. Path 3 features policy support and teacher professional competence but lacks adequate organizational leadership, resource allocation, student experience, curriculum implementation, facilities, and evaluation mechanisms. Path 6 possesses policy support and curriculum implementation conditions but is weak in other organizational and participatory aspects. Path 8 includes resource allocation and evaluation mechanisms but falls short in organizational leadership, student experience, curriculum implementation, and teacher-related conditions. These results indicate that the mere presence of certain external conditions does not automatically yield high quality; external support must be translated into practice through organizational coordination, curriculum delivery, teacher implementation, and student engagement—findings that support Proposition 4 (P4).

P3 and P4 are not simply the inverse of high-quality paths. Insufficient foundational support highlights a school's lack of the basic conditions required to sustain regular physical education activities, whereas a failure of organizational transformation emphasizes that existing conditions have not been successfully integrated into daily educational practice. Consequently, these two scenarios require distinct governance and improvement strategies.

### 5.4 Robustness and sensitivity tests

Robustness tests varied the full-membership anchor, consistency threshold, PRI threshold, case weight, frequency threshold, variable operationalization, random subsample, and model specification. The analysis examined changes in path number, consistency, coverage, and core-condition stability.

**Figure 8:**
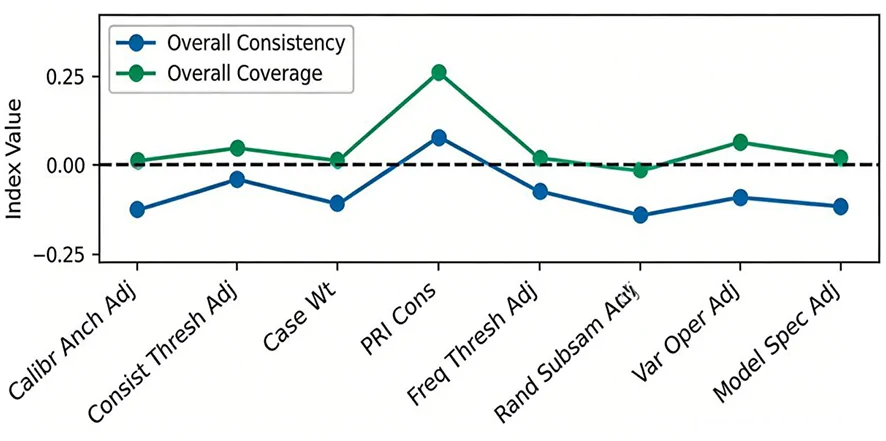
Comparative analysis of robustness and sensitivity results.

Raising the calibration anchor from 0.80 to 0.85 changed overall consistency by 0.012 and coverage by −0.008 without altering the number of paths. Lowering the consistency threshold added one path and retained a stability index of 0.88. Lowering the PRI threshold added two marginal paths. Changing variable operationalization reduced consistency by 0.025, increased coverage by 0.020, and retained a stability index of 0.81. Figure 8 and Table 9 report the full results.

**Table 9:** Robustness and sensitivity test results.

Test parameters	Original setting	Adjusted	Consistency change	Change in coverage	Path change	Stability index	Case consistency	Simplicity change
Calibration anchor point	0.8	0.85	0.012	−0.008	0	1	1	0.03
Consistency threshold	0.85	0.8	−0.018	0.015	1	0.88	0.94	−0.05
Case weight	1	0.9	0.008	−0.006	0	1	1	0.02
PRI consistency	0.75	0.7	−0.015	0.012	2	0.75	0.89	−0.08
Frequency threshold	1	2	0.022	−0.018	−1	1	1	0.06
Random subsample	1	0.9	± 0.010	± 0.008	±1	0.94	0.97	± 0.04
Variable operationalization	1	0.95	−0.025	0.02	1	0.81	0.92	−0.07
Model specification	1	1.05	0.015	−0.012	0	1	1	0.03

The main explanatory framework remained broadly stable across the tested settings, although looser thresholds introduced marginal paths and variable operationalization affected several statistics. Robustness therefore refers to stability within the examined parameter range, not immunity to analytical choices. These tests strengthen internal credibility but do not remove the limitations of a five-case design.

## 6 Discussion and conclusion

### 6.1 Research conclusions and main contributions

This study explains variations in the quality of school physical education activities through the lens of cross-level transformation. The results indicate that securing policy and resource support does not automatically translate into high-quality outcomes; external conditions must be converted into actual educational results through organizational leadership, teacher implementation, curriculum delivery, evaluation and feedback, and student participation. Case studies and configurational analysis across five schools reveal four distinct mechanisms: resource transformation, organizational compensation, failure of foundational support, and failure of organizational transformation.

Regarding high-quality outcomes, the resource transformation mechanism (P1) is supported by the case data. Schools with relatively abundant resources can only translate their advantages in funding, staffing, and facilities into high-quality activities if they also possess strong organizational coordination, effective curriculum implementation, and high teacher professional competence. The organizational compensation mechanism (P2) also receives exploratory support; where certain external conditions are lacking, strong organizational capabilities, effective curriculum implementation, teacher collaboration, and positive student experiences can enhance the utilization efficiency of existing resources.

In the context of low-quality outcomes, the failure of foundational support mechanism (P3) manifests as multiple deficiencies across policy, resources, teaching staff, facilities, and curriculum conditions. The failure of organizational transformation mechanism (P4) demonstrates that even when schools possess some policy or resource advantages, low-quality outcomes can still arise if organizational coordination, curriculum execution, evaluation and feedback, and student participation are not effectively activated. These two failure mechanisms illustrate that increasing inputs and enhancing transformation capabilities represent distinct governance tasks.

The contribution is not a general restatement of causal complexity but a school-level account of how external support, organizational processes, and student experience interact. The four propositions remain exploratory because they are based on five schools.

The analysis distinguishes resource transformation from organizational compensation. Resource-driven schools require effective conversion of funding, teachers, and facilities into educational benefits, whereas organizationally innovative schools can partly offset moderate resources through coordination, curriculum improvement, and student engagement.

It also treats low quality as an independent mechanism. Failure may reflect broad foundational deficiencies or the inability to convert existing support into practice. This asymmetric framework offers propositions for testing in larger and longitudinal samples rather than a universal school typology.

### 6.2 Research limitations and future directions

The first limitation is the five-school case base, which cannot represent all combinations of eight antecedent conditions. Maximum-variation sampling improves theoretical contrast but not statistical coverage. Path overlap and logical remainders may therefore affect interpretation. The reported configurations should be treated as exploratory, and future studies should test their recurrence across larger samples, regions, and school levels.

The second limitation concerns measurement. The organizational leadership and student experience scales received preliminary support from expert review, reliability, and convergent validity but require validation across wider school populations. Other school-level indicators may be affected by incomplete records or differences in judgment. Future work should test scale structure and discriminant validity and incorporate third-party evaluation, behavior records, and process data.

Third, common method bias may still exist because several variables were collected within the same research process. This study attempted to reduce this risk by using multiple data sources, including questionnaires, interviews, field observations, archival materials, and expert review. Nevertheless, future studies should further separate data sources, collection time, and respondent groups, so that the measurement of condition variables and outcome variables can be more clearly distinguished.

Fourth, the regional scope of this study is limited. The selected cases reflect certain differences among eastern, central, and western school contexts, but they cannot fully represent all school sports development situations. Differences in local policy implementation, fiscal capacity, school governance culture, family sports support, and community sports resources may influence the formation of sports activity quality. Future research should further include schools from more diverse social and cultural settings.

Finally, this study is mainly based on cross-sectional data, which makes it difficult to fully capture the dynamic process of quality formation. School sports activity quality may change over time as policies, resources, school leadership, teacher teams, and student participation patterns change. Future research can adopt longitudinal designs to observe how different configurations evolve and how schools move from low-quality to high-quality development paths.

### 6.3 Practical insights: from “Best Practices” to “Precise Matching”

Practice should move from uniform best-practice prescriptions to precise matching. Resource-abundant schools should prioritize conversion efficiency through curriculum design, teacher collaboration, quality control, and student feedback. Schools with moderate resources should strengthen coordination, flexible scheduling, teacher motivation, and family-community cooperation. Resource-constrained schools should first secure minimum curriculum delivery, safe activity space, teacher support, and feasible local programs, then select one or two realistic breakthrough points.

Educational authorities should provide differentiated support, while schools use self-diagnosis to identify their position and improvement path. Teachers need stronger resource-integration and instructional-innovation capacity. Sustainable improvement depends on coordination among policy guidance, school management, teacher implementation, and student participation.

## Data Availability

The raw data supporting the conclusions of this article will be made available by the authors, without undue reservation.
