# Correction: Heterogeneity manifestation and formation mechanism of school youth sports activity quality: a multi-case comparative study based on the configurational perspective

**DOI:** 10.3389/fpsyg.2026.1985900

**Published:** 2026-09-17

**Authors:** Yang Liu, Jiesen Yin

**Affiliations:** 1 Department of Physical Education, Changzhou Vocational Institute of Textile and Garment, Changzhou, Jiangsu, China; 2 Department of Physical Education, Wuxi University of Technology, Wuxi, Jiangsu, China

**Keywords:** configuration analysis, exact match, formation mechanism, quality heterogeneity, youth sports

An incorrect Funding statement was provided. The correct funding statement reads:

The author(s) declared that financial support was received for this work and/or its publication. This work was supported by the Ministry of Education of China Humanities and Social Sciences Research Planning Fund Project (Grant No. 25YJAZH214), titled “An Empirical Study on the Evaluation System for Adolescents' Physical Activity Quality from the Perspective of Building a Strong Educational Nation”; the “Qinglan Project” of Jiangsu Universities (2024) (Su Shi Han [2024] No. 14); the Jiangsu Province's 14th Five-Year Plan Key Educational Research Project (Grant No. B/2022/01/25); and the Jiangsu Province Higher Education Special Project on Teaching Reform of College Students' Quality Education Courses (Grant No. 2025DSZ099).

The original version of this article has been updated.

